# Aphasia as the Initial Presentation of Acute Otomastoiditis in an Elderly Male

**DOI:** 10.7759/cureus.14293

**Published:** 2021-04-05

**Authors:** Sherif T Abuserewa, Mohamed Faris, Thaddeus Golden

**Affiliations:** 1 Department of Internal Medicine, Grand Strand Regional Medical Center, Myrtle Beach, USA; 2 Department of Pulmonary and Critical Care Medicine, Grand Strand Regional Medical Center, Myrtle Beach, USA

**Keywords:** global aphasia, otomastoiditis, intracranial epidural abscess, cerebellopontine angle abscess

## Abstract

Acute otomastoiditis is a potentially life-threatening condition. It is relatively uncommon in elderly patients and is usually seen as a complication of acute otitis media. Limited studies are available on this uncommon disease in the elderly, and thus the clinical course of the disease is not fully understood. Proper use of antibiotics for acute otitis media has markedly decreased the incidence of acute otomastoiditis. We describe a case of a 76-year-old male with acute otomastoiditis complicated by cerebellopontine angle epidural abscess who presented with a chief complaint of sudden onset of global aphasia, which is an unusual presentation for acute otomastoiditis. The patient eventually required medical and surgical intervention. To the best of our knowledge, acute otomastoiditis presenting with sudden onset of global aphasia has not been previously described in the literature. This case illustrates how multidisciplinary team involvement is vital in the management of these uncommon cases.

## Introduction

Acute mastoiditis is one of the most common intracranial complications of acute otitis media due to the close anatomical position of the ear and the mastoid bone, and this proximity facilitates the spread of infection [1]. Acute mastoiditis is known to be more common in children than in adults. However, its incidence has markedly decreased with the routine use of antibiotics for the treatment of otitis media. Acute mastoiditis is now rarely seen in elderly people. As a result of this decrease in incidence, physicians might not be as experienced or aware of how to treat and diagnose this complication [2]. In this case, we will show an uncommon presentation for otomastoiditis and how involvement of a multidisciplinary team was able to achieve early diagnosis and prompt treatment with favorable outcome in this unusual complicated case.

## Case presentation

A 76-year-old gentleman with a past medical history of hypertension, type 2 diabetes mellitus, and coronary artery disease with coronary artery bypass graft in 2006 presented with a sudden onset of inability to speak. His wife reported that the day prior to his admission to our facility, he was complaining of flu-like symptoms. He drove himself to his primary care physician on the morning of admission and was prescribed amoxicillin. A few hours later, she found him on the floor. He was aphasic at that point.

Upon arriving to our emergency department, the patient had an altered mental status with a Glasgow coma scale of 12, his vitals showed a temperature of 100.8°F, heart rate of 120 beats per minute, and a respiratory rate of over 20 breaths per minute, and his oxygen saturation was 82% on room air. He had global aphasia with incomprehensible sounds as he was not following commands and produced incomprehensible sounds. Neurological examination showed sluggishly reactive, equal pupils 2 mm in diameter. Oculocephalic reflex was present. Corneal reflex was intact and so was the gag reflex. Motor examination showed generalized weakness, and deep tendon reflexes were absent throughout. Sensory exam showed no deficits. Coordination and gait could not be assessed due to the patient's condition and lack of cooperation with examination. Otological examination showed well-formed external auricles bilaterally, and there was no right-sided post-auricular erythema or fluctuance. Right external auditory canal was patent with moderate cerumen impaction, and the tympanic membrane was intact with thickening and evidence of fluid in the middle ear. There was no evidence of cholesteatoma or retraction pocket, and malleus and incus could not be visualized. No neurological focal deficit was noted. Chest auscultation revealed fine crackles on the left inter-scapular and infra-scapular zones.

Pertinent findings on laboratory tests included leukocytosis with a white cell count of 23.6 K/mm^3^ (87.5% PMNL [polymorphonuclear leukocyte]), an elevated procalcitonin of 15 ng/mL, lactic acidosis, creatinine 1.78 mg/dL with a GFR (glomerular filtration rate) of 37 mL/min/1.73m^2^ with no known history of chronic kidney disease, a mildly elevated troponin of 0.208 ng/mL, elevated pro-BNP (proB-type natriuretic peptide) at 3,500 pg/mL, and a urinalysis negative for infection. Electrocardiography showed sinus tachycardia with a right bundle branch block. Blood, urine, and sputum cultures were drawn. The patient underwent multiple imaging studies; chest X-ray showed left lower lobe heterogeneous opacity and consolidation, a computed tomography (CT) scan of the head did not show any acute abnormalities, and a CT angiography of the head and neck showed suspicion of right-sided otomastoiditis with intracranial extension. Bilateral lower extremity venous duplex scans were negative.

The patient met sepsis criteria and was therefore given the appropriate sepsis bundle including intravenous fluids and broad-spectrum antibiotics (vancomycin, cefepime, and metronidazole). He was placed on non-invasive mechanical ventilation (BiPAP) and was admitted to the intensive care unit.

**Figure 1:**
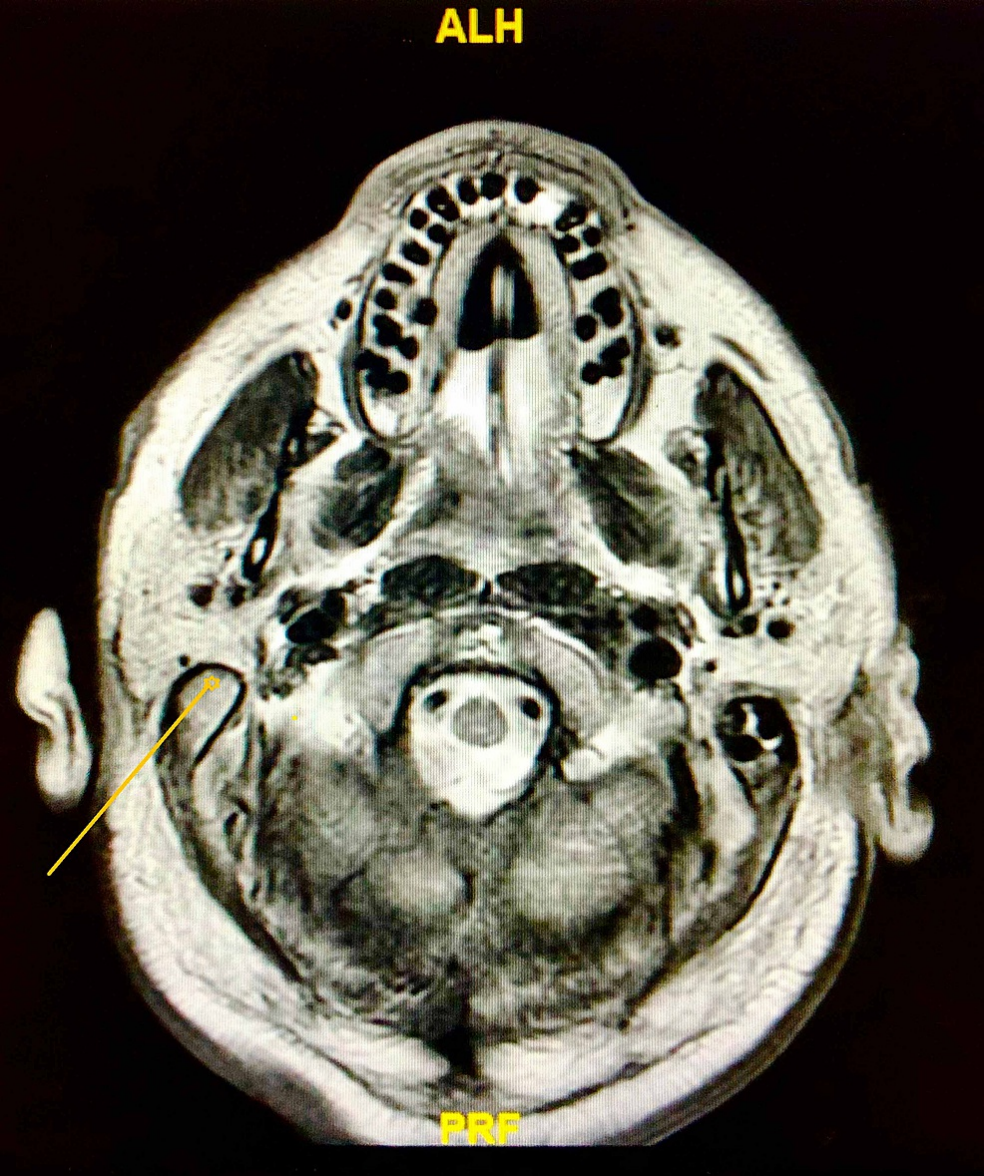
Brain MRI showing evidence of right-sided otomastoiditis (yellow arrow)

**Figure 2:**
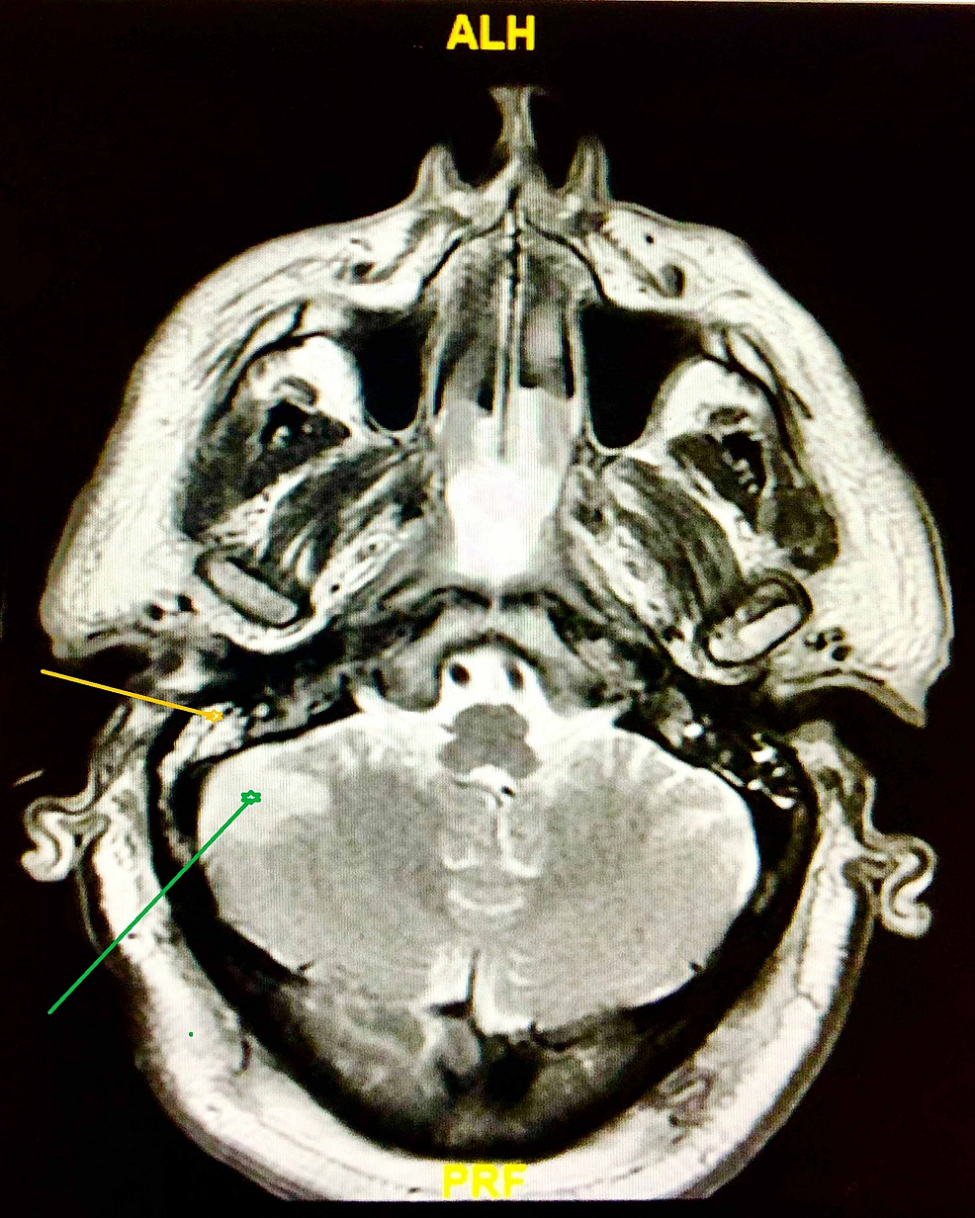
: Brain MRI showing evidence of right-sided otomastoiditis (yellow arrow) with intracranial extension and cerebellopontine epidural abscess (green arrow)

The lactic acidosis resolved with the administration of intravenous fluids boluses. However, the patient’s hypoxia worsened rapidly and intubation with mechanical ventilation was necessary. Magnetic resonance imaging (MRI) of the brain was ordered, which showed evidence of right-sided otomastoiditis with intracranial extension (as shown in Figure 1), cerebellopontine angle epidural abscess, and possible cerebral abscess (as shown in Figure 2); FLAIR (fluid-attenuated inversion recovery) signal abnormality was noted in the sulci of the frontal lobes, and restricted diffusion was noted in the sulcus of the right parietal occipital junction, which was concerning for developing meningitis. Otorhinolaryngology team was consulted and the patient underwent myringotomy with middle ear pus drainage followed by myringotomy tube placement.

Otic ciprofloxacin drops were started, and the patient was taken to the operating room for mastoidectomy with drainage of epidural abscess. Blood, urine, sputum, and ear drainage cultures did not show bacterial growth. The patient deteriorated and developed septic shock. Infectious disease service was consulted, and recommendations were made to keep the same antibiotic regimen as mentioned previously. A repeat MRI of the brain showed residual abscess. Neurosurgery service was consulted and proceeded to perform a right sub-occipital craniectomy. No frank purulence was noted. Recommendations were made to keep the patient on the antibiotics for three weeks after the neurosurgical intervention. The patient finally became stable and was extubated and transferred out of the intensive care unit after a 14-day stay.

## Discussion

This is a case of complicated otomastoiditis in an elderly male who presented with sudden onset of global aphasia secondary to cerebellopontine angle epidural abscess. In the United States, the incidence of mastoiditis is low and estimated at four per 100,000. The incidence is much lower in the elderly population [3]. The incidence of intracranial complications of otomastoiditis has become rare in the era of effective antibiotics, which is estimated to be 0.1-2%, and similarly, the mortality rate has markedly declined from 35% to 5% [4].

Acute mastoiditis is defined as inflammation of the mastoid air cells, which can lead to accumulation of purulent effusion and destruction of the bony trabeculae leading to a single common cavity. It may extend into different anatomical areas resulting in multiple different complications such as labyrinthitis, Bezold’s abscess (deep neck abscess secondary to erosion of the cortex by acute otomastoiditis infection medial to the attachment of sternocleidomastoid and extending into the infratemporal fossa), petrositis, Gradenigo's syndrome (extension of mastoiditis to the apex of the petrous temporal bone leading to the triad of suppurative otitis media, pain in the distribution of the trigeminal nerve, and abducens nerve palsy), and intracranial abscesses. Intracranial abscesses can result from the spread of infection in the middle ear or mastoid either hematogenously or directly from adjacent structures. This can lead to petrositis, meningitis, or lateral sinus thrombophlebitis [5].

Mastoiditis in adults may present either acutely or gradually. Elderly patients in particular can be at risk of rapid deterioration especially with intracranial complications. Therefore, these patients should be admitted to the hospital and started on intravenous antibiotics promptly. Once mastoiditis is suspected, a CT scan of the head should be ordered. If there is any suspicion of an intracranial complication, then an MRI of the brain should be considered to confirm the diagnosis.

Mastoiditis may present with fever, posterior ear pain, local erythema over the mastoid bone, edema of the ear pinna, or posterior and downward displacement of the auricle. Our case is unique in that the patient presented with global aphasia. Initially, the case was suspected to be a cerebrovascular accident. However, the brain imaging completely changed the suspected diagnosis. The patient is right-handed and therefore his speech center is most likely in the left hemisphere, which was the non-affected side. Brain imaging did not show any evidence of cerebritis or brain abscess in the left hemisphere or any sinus venous thrombosis that may cause aphasia. Cerebellopontine angle epidural abscess presenting with aphasia has been reported in the literature [6]. The presence of purulent fluid in the posterior fossa may also result in cerebral vasospasm that may affect the speech center. Cerebral vasospasm may occur with cerebral inflammation and bacterial infections due to the release of superoxide anions, tumor necrosis factor (TNF), and interleukins (ILs) causing inactivation of nitric oxide (NO) and expression of endothelin-1 (ET-1) causing vasospasm.

The most common site of a brain abscess due to an otogenic source is the temporal lobe and cerebellum, which occurred in this case. Several organisms have been reported in the literature to be a possible causative agent. Of these, *Staphylococcus aureus*, *Streptococcus pneumoniae*, *Haemophilus influenzae*, *Pseudomonas*, *Proteus*, and *Escherichia coli* were the most commonly reported organisms [7-9].

Surgical treatment in addition to broad-spectrum antibiotics is the most effective treatment reported for a brain abscess. Conservative management may be considered in some cases, for example, in multiple or inaccessible brain abscesses or in high-risk surgical patients [10].

Aphasia is the difficulty or loss of ability to speak or understand (or both) of spoken or written language or both. The main language center comprises Broca's area in the posterior inferior frontal gyrus controlling the spoken language, Wernicke's area that is the posterior two-thirds of the superior temporal gyrus responsible for assigning words meaning, and the angular gyrus in the inferior parietal lobule responsible for perception of written language [11]. Some other areas contribute to language processing including the insula responsible for articulation [12], multiple frontal and temporal lobe areas for sentence processing [13], and several areas in temporal, occipital, and parietal cortex for words, knowledge, and meanings [14]. Left-hemisphere is dominant for language function in more than 95% in right-handed people [15] and in 30-70% of left-handed people [16].

Aphasia can be categorized into different types and aphasia syndromes. Global aphasia is the one which was focused on in this case. Global aphasia includes deficits in all language functions either expressive or receptive [17]. Patients usually do not follow commands and produce incomprehensible sounds or non-word utterances. Generally, patients have right neurological deficits including right hemiparesis or right visual field deficit [17,18]. However, global aphasia is documented in the literature without lateralizing signs in case of encephalitis, late stage of dementia, and lesions involving Broca’s and Wernicke’s areas selectively [19].

It is very interesting to know that patients with metabolic encephalopathy or delirium may present with symptoms and signs mimicking global aphasia, including difficulty in naming objects and inability to follow commands [20]. It will differ from global aphasia in being associated with fluctuation of mentation, hallucinations, or asterixis [20].

It is really hard to confirm in the presented case if the patient had global aphasia or just metabolic encephalopathy mimicking global aphasia in presentation as the patient did not have any other neurological deficits and the site of the lesion is uncommon to affect language centers. However, given that the patient was left-handed, this is bringing up a possibility for the presence of language centers in the right hemisphere. Moreover, the wide distribution of areas involved in language processing, as discussed above, and mildly improved unresolved aphasia after the almost complete resolution of sepsis are favoring the diagnosis of global aphasia more than just metabolic encephalopathy.

## Conclusions

Otomastoiditis in the elderly is a rare diagnosis nowadays. It presents with a classical clinical picture in about 50% of cases. It is associated with a relatively high risk of complications and mortality. Global aphasia can be a presenting symptom for otomastoiditis with intracranial extension regardless of the presence of metabolic encephalopathy, which can mimic global aphasia. Limited descriptions of otomastoiditis in the elderly are available, and thus the clinical course of the disease is not fully understood. Clinicians need to have a high suspicion to make an early diagnosis and start prompt management to avoid life-threatening complications.
